# No benefit of the trochanteric stabilizing plate on loss of fracture reduction in AO/OTA 31-A2 trochanteric fractures: a randomized controlled trial using radiostereometric analysis

**DOI:** 10.1302/2633-1462.51.BJO-2023-0082.R1

**Published:** 2024-01-19

**Authors:** Carl E. Alm, Anders Karlsten, Jan E. Madsen, Lars Nordsletten, Jan E. Brattgjerd, Are H. Pripp, Frede Frihagen, Stephan M. Röhrl

**Affiliations:** 1 Division of Orthopaedic Surgery, Oslo University Hospital, Oslo, Norway; 2 Institute of Clinical Medicine, Faculty of Medicine, University of Oslo, Oslo, Norway; 3 Lovisenberg Diaconal Hospital, Oslo, Norway; 4 Faculty of Health Sciences, Oslo Metropolitan University, Oslo, Norway; 5 Oslo Centre of Biostatistics and Epidemiology, Research Support Services, Oslo, Norway; 6 Department of Orthopaedic Surgery, Østfold Hospital Trust, Grålum, Norway

**Keywords:** hip, trochanteric fractures, sliding hip screw, trochanteric stabilizing plate, radiostereometric analysis, fracture healing, stability, Trochanteric fractures, fracture reduction, radiostereometric analysis (RSA), post operative fracture, clinical outcomes, femoral shaft, biomechanical studies, radiographs, femoral head

## Abstract

**Aims:**

Despite limited clinical scientific backing, an additional trochanteric stabilizing plate (TSP) has been advocated when treating unstable trochanteric fractures with a sliding hip screw (SHS). We aimed to explore whether the TSP would result in less post operative fracture motion, compared to SHS alone.

**Methods:**

Overall, 31 patients with AO/OTA 31-A2 trochanteric fractures were randomized to either a SHS alone or a SHS with an additional TSP. To compare postoperative fracture motion, radiostereometric analysis (RSA) was performed before and after weightbearing, and then at four, eight, 12, 26, and 52 weeks. With the “after weightbearing” images as baseline, we calculated translations and rotations, including shortening and medialization of the femoral shaft.

**Results:**

Similar migration profiles were observed in all directions during the course of healing. At one year, eight patients in the SHS group and 12 patients in the TSP group were available for analysis, finding a clinically non-relevant, and statistically non-significant, difference in total translation of 1 mm (95% confidence interval -4.7 to 2.9) in favour of the TSP group. In line with the migration data, no significant differences in clinical outcomes were found.

**Conclusion:**

The TSP did not influence the course of healing or postoperative fracture motion compared to SHS alone. Based on our results, routine use of the TSP in AO/OTA 31-A2 trochanteric fractures cannot be recommended. The TSP has been shown, in biomechanical studies, to increase stability in sliding hip screw constructs in both unstable and intermediate stable trochanteric fractures, but the clinical evidence is limited. This study showed no advantage of the TSP in unstable (AO 31-A2) fractures in elderly patients when fracture movement was evaluated with radiostereometric analysis.

Cite this article: *Bone Jt Open* 2024;5(1):37–45.

## Introduction

Trochanteric fractures with posteromedial comminution or loss of lateral wall integrity are deemed unstable,^1^ and remains a challenge to both surgeons and patients with high rates of failure and functional impairment.^2,3^ Implants with a lag screw allowing fracture compression during healing seems to be favourable compared to fixed angle devices in trochanteric fractures.^4,5^ However, particularly in unstable fracture patterns, this will result in fracture subsidence to some degree. A correlation between shortening and altered gait in patients with surgically treated trochanteric fractures has been reported.^3,6^ In consequence, any measure that could enhance fracture healing and reduce secondary fracture displacement would be of benefit.

We sought to explore whether the trochanteric stabilizing plate (TSP) would reduce postoperative fracture movement compared to the sliding hip screw (SHS) alone. The clinical evidence in support of the TSP is scarce.^7^ No significant difference between SHS with or without TSP was found in the only randomized controlled trial (RCT) published.^8^ Still, the TSP is frequently used in several parts of the world.^9-12^

The failure mode of trochanteric fractures is previously shown to be multidirectional,^13,14^ often including a substantial secondary displacement in rotation. While evaluation and quantification of rotation is difficult using conventional radiographs, radiostereometric analysis (RSA) performed over time enables accurate analysis of movement in both translation and rotation. This makes it feasible when comparing the course of healing, failure modes, and mechanical properties of different fixation methods or implants^15-17^

The aim of this study was to evaluate the effect of the TSP in a RCT comparing secondary fracture displacement, measured by RSA, in unstable trochanteric fractures (AO/OTA 31-A2)^18^ operated with SHS with or without an additional trochanteric stabilizing plate.

## Methods

**Fig. 1:**
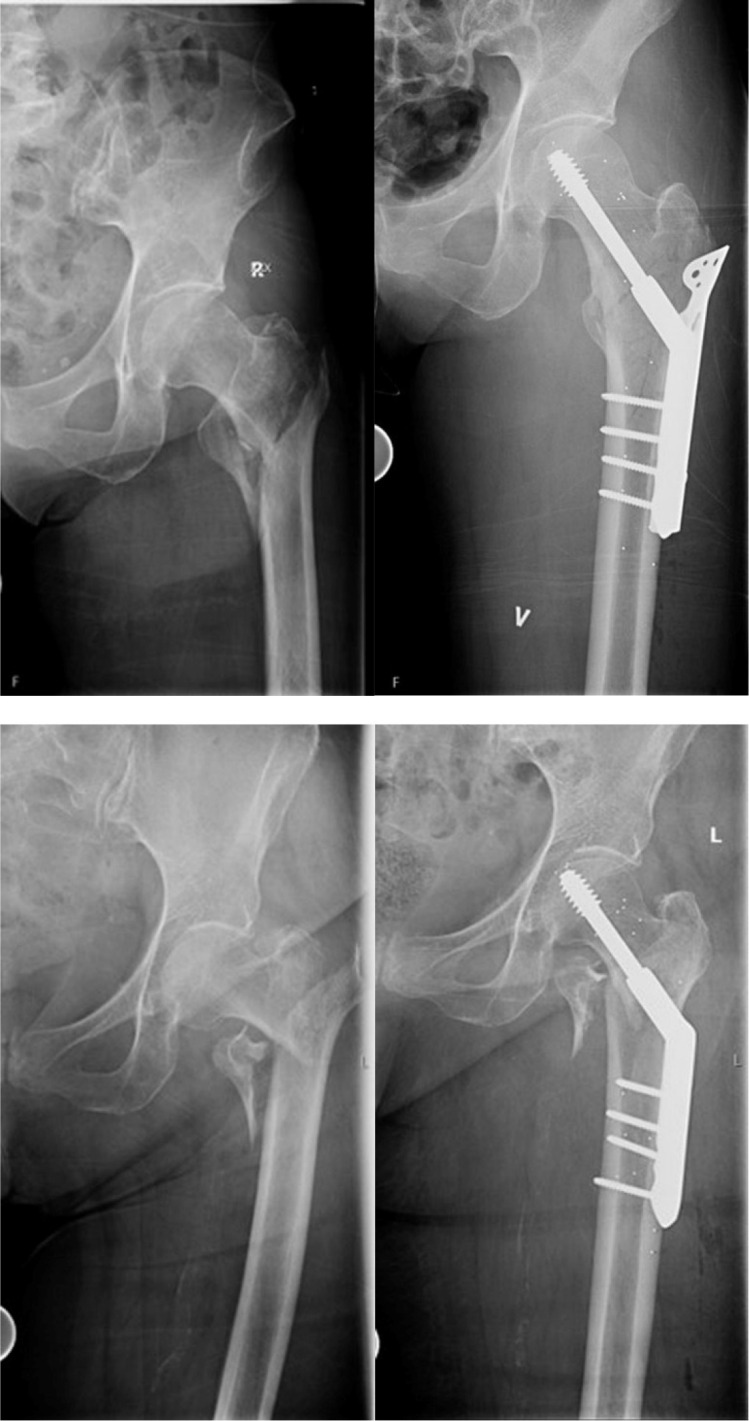
Examples of included AO/OTA 31-A2 fractures. Pre- and postoperative radiographs.

Between November 2014 and December 2016, 31 patients with AO/OTA 31-A2 trochanteric fractures (Figure 1) were enrolled in the study after providing written informed consent. Our inclusion criteria were AO/OTA type 31-A2 trochanteric fracture, aged over 50 years, able to walk independently with or without aids, able to consent, and fit for surgery. Patients not willing or able to attain follow-up, with previous fracture in the same hip or concomitant disease that could shorten life expectancy, e.g. end stage cancer, were excluded from the study. All participants were recruited and operated at Ullevål University Hospital and Diakonhjemmet Hospital, Norway. Eligible patients were enrolled by the orthopaedic surgeon responsible on each site (CEA, AK). The patients were allocated to treatment group by a computer-generated sequence with variable block sizes and stratification on hospital. Sealed sequentially numbered opaque envelopes were made for each hospital. The envelopes were opened in the operating room. Follow-up examinations took place at the hospital where the surgery was performed.

### Surgical technique

All patients, in both groups, were operated by the first (CEA) or second author (ARK) in a standardized manner. A traction table was used to obtain fracture reduction and a lateral approach was performed. The guide wire for the lag screw was inserted using the 135° guide, aiming for the center-center position in the femoral head.^19^ After confirmation of wire placement and screw length measurement, preparation for the barrel and lag screw were performed with the DHS triple reamer. The fractures were fixed using the LCP DHS (DePuy Synthes, USA) with or without a TSP (DePuy Synthes) depending on the randomization.

### RSA

Using a specially designed insertion device (UmRSA; RSA Biomedical, Sweden), six to eight tantalum markers (1 mm) were implanted in the femoral head through the reamed canal for the sliding screw and/or through the fracture gap, while markers in the femoral shaft were implanted through the lateral and anterior cortex using drill holes or a surgical awl (Figure 1).

RSA images were obtained before weightbearing, before discharge from the hospital, and then at scheduled follow-ups after four, eight, 12, 26, and 52 weeks postoperatively. The RSA examinations were executed in the same manner in both hospitals with a similar setup. With the patient supine and a uniplanar calibration cage no 43 (UmRSA; RSA Biomedical) underneath the examination table, all images were obtained by a trained radiographer. At Oslo University Hospital, ceiling-mounted radiograph tubes were used, while a combination of ceiling and portable tubes were used at Diakonhjemmet Hospital.

UmRSA (digital measurement 6; RSA Biomedical) was used for all analyses of movement along the global coordinate system. The femoral shaft was defined as the fixed segment and the head/neck fragment as the moving segment. As a measure of tantalum marker distribution, a condition number (CN) was calculated for each RSA examination. In accordance with Valstar et al,^20^ RSA images with condition numbers above 150 in either segment were discarded from the analysis. Mean error (ME) of rigid body fitting of less than 0.35 mm was accepted. For the precision analysis, dual examinations were performed on the same day with the patient and the radiograph set up repositioned in between examinations. Assuming no fracture movement between the examinations, the precision was calculated by multiplying the standard deviation (SD) in each direction with the critical value (1.98) obtained from the T-table adjusted for the number of dual examinations minus 1. The images obtained after the first weightbearing were chosen as baseline since ten patients were missing immediate postoperative RSA images before mobilization. Including patients with images both before and after weightbearing a separate analysis was performed to compare migration between the two treatment groups.

Conventional radiographs (pelvic and lateral) were taken at admission, postoperatively, and after four months. The lateral wall thickness was calculated from the preoperative images as described by Hsu et al.^21^ The tip to apex distance (TAD) was calculated after calibration of the images using the known diameter of the sliding screw and the reduction quality was analyzed from the postoperative radiographs.^22^

At each scheduled follow-up, EuroQol five-dimension questionnaire (EQ-5D),^23^ Harris Hip Score,^24^ Timed Up and Go (TUG) test,^25^ pain (Numeric Rating Scale (NRS))^26^ and Satisfaction with operated hip (NRS) were obtained. Surgical and medical complications were registered continuously.

The primary outcome measure in the study was migration of the femoral head/neck fragment relative to the shaft, measured by RSA, expressed as total translation (t3D) at one year. Secondary outcomes were translation and rotation around the X-, Y, and Z-axis at all time points. Even though the study was not powered to show differences in the patient reported outcomes, these were included as exploratory outcome measures. The conventional radiograph measurements were performed to control for factors that might influence fracture fixation stability.

### Statistical analysis

Our primary end point was movement during healing as measured by RSA. In the absence of a well-defined clinically relevant threshold in the literature, we decided on a difference in total translation of 2 mm when calculating sample sizes. A difference of 2 mm might be clinically relevant and is well above the accuracy of the chosen method (RSA) for evaluation of our primary outcome. With a statistical power of 80% and a significance level of 5%, and with an expected precision of 0.3 mm^15^, we calculated a need for eightt patients in each group. Since RSA rarely has been used in geriatric fracture models, we increased the number of participants to 15 in each group to encounter for precision estimate uncertainty and expected problems with inferior radiographs, and loss to follow-up.

Baseline demographics (sex, age, American Society of Anesthesiologists (ASA) score), perioperative data (duration of surgery, blood loss) and conventional radiograph measurements (side, lateral wall thickness, TAD, reduction quality) are presented as mean or median, values with 95% confidence interval (CI), or as frequencies and percentages as appropriate. To calculate RSA migration and clinical outcome scores, a linear mixed model (LMM) with a subject-specific random intercept and main and interaction fixed effects of time and group was used. The LMM is preferred in cases of repetitive measurements on the same individuals as it better accommodates missing data than simpler statistical models.^27^ All available data at any time point were included in the analyses. Additional linear mixed model analysis was conducted to control for baseline imbalances. Migration and clinical data is presented as mean values with 95% CIs. A p-value < 0.05 was deemed statistically significant. All patients were treated according to their randomization group.

Analyses of baseline demographics, perioperative data and conventional radiograph measurements were performed with SPSS Statistics for Windows, v.27 (IBM, USA). Stata version 17 (StataCorp, USA) were used for the analysis of RSA and clinical data.

### Ethics, registration, funding, and potential conflict of interests

The study protocol was approved by the Regional Ethics Committee South-East Norway B (2014/475) and registered at Clinicaltrials.gov (NCT02294747). Prior to surgery, all patients provided written informed consent and the study was conducted in accordance with the Helsinki Declaration. No external funding was received. No conflicts of interest are reported by the authors.

## Results

**Fig. 2:**
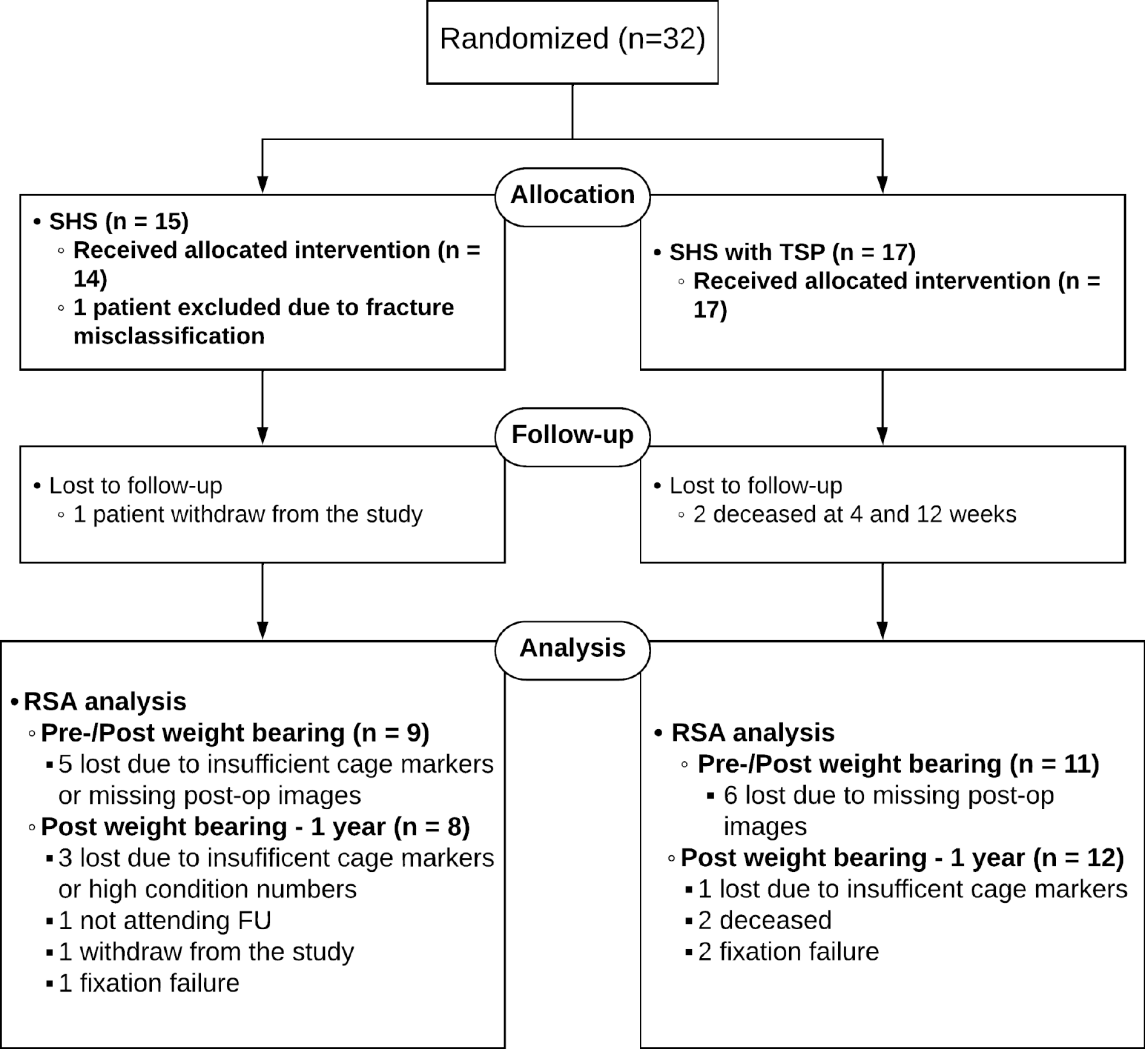
Consort flowchart. FU, follow-up; SHS, sliding hip screw; TSP, trochanteric stabilizing plate; RSA, radiostereometric analysis,

In total, 32 patients were included in the study (Figure 2). One patient in the SHS group was excluded after randomization due to misclassification of the fracture. Overall, 14 patients were operated with SHS alone while 17 patients received an additional TSP. There were more males, and the patients were younger in the SHS group (Table I). Otherwise, the randomization groups were reasonably balanced. The baseline disbalance did not alter the primary or secondary outcome measures as assessed by linear regression (Supplementary Table i).

**Table I.** Patient demographics, surgery and fracture characteristics, hardware failure and mortality rates.

Variable	SHS	SHS with TSP
Total, n	14	17
Female, n (%)	9 (64)	16 (94)
Median age, yrs (range)	79 (67 to 87)	88 (62 to 94)
**ASA class, n (%)**		
1 to 2	7 (50)	12 (71)
3 to 4	7 (50)	5 (29)
Left side, n (%)	7 (50)	10 (59)
Mean lateral wall thickness, mm (95% CI)*	22.1 (18.3 to 26.0)	20.5 (17.7 to 23.2)
Mean TAD, mm (95% CI)†	20.1 (15.6 to 24.5)	17.1 (13.6 to 20.6)
**Reduction quality, n** ‡		
1	9	9
2 to 3	5	8
Mean perioperative bleeding, ml (95% CI)	293 (220 to 366)	292 (183 to 401)
Mean duration of surgery, mins (95% CI)	75 (54 to 96)	74 (56 to 93)
Fixation failure, n	1	2
Mortality, n		2

* Lateral wall thickness measured from the preoperative images, as described by Hsu et al.^21^

† Calculated as described by Baumgaertner et al.^22^ Images calibrated against known sliding screw diameter.

‡ Described by Baumgaertner et al.^22^ 1 = good (normal or slight valgus in anteroposterior view, < 20° of angulation in lateral view and < 4 mm displacement); 2 = acceptable (good reduction with respect to either alignment or displacement); 3 = poor (neither criterion met).

ASA, American Society of Anesthesiologists; CI, confidence interval; SHS, sliding hip screw; TAD, tip to apex distance; TSP, trochanteric stabilizing plate.

The precision, calculated from 106 dual examinations, was acceptable for both translation and rotation (Table II).

**Table II.** Precision in translation and rotation based on 106 dual examinations.

Variable	SD	Precision*
**Fracture translation, mm**		
X-axis (medial/lateral)	0.12	0.24
Y-axis (proximal/distal)	0.13	0.26
Z-axis (anterior/posterior)	0.15	0.30
3D (t3D)	0.21	0.42
**Fracture rotation, degrees**		
X-axis (anterior/posterior)	0.23	0.47
Y-axis (ante-/retroversion)	0.77	1.53
Z-axis (varus/valgus)	0.28	0.56

* Assuming no fracture movement between the examinations, the precision was calculated by multiplying the standard deviation in each direction with the critical value (1.98) obtained from the T-table adjusted for the number of dual examinations minus 1.

SD, standard deviation.

Mean total translation (t3D) after the first weightbearing was 3.5 mm (95% CI 0.63 to 6.36) in the SHS group and 3.4 mm (95% CI 1.00 to 5.89) in the TSP group (Table III).

**Table III.** Migration analysis of femoral head/neck movement relative to the shaft pre- and post-weightbearing ("week 1"), and from after weightbearing ("week 1") to one year postoperatively.

Variable	SHS (n = 9)	SHS with TSP (n = 11)	Mean difference	p-value*
**Pre-/post-weightbearing**				
**Fracture translation, mm (95% CI)**				
X-axis (+ medial/- lateral)	1.23 (-3.01 to 0.54)	1.18 (-2.70 to 0.33)	0.05 (-2.28 to 2.38)	0.967
Y-axis (+ proximal/- distal)	3.09 (-5.48 to -0.70)	2.72 (-4.76 to -0.69)	0.36 (-2.78 to 3.50)	0.821
Z-axis (-anterior/+ posterior)	0.75 (-1.90 to 0.40)	0.50 (-1.46 to 0.47)	0,25 (-1.23 to 1.74)	0.737
Total translation (t3D)	3.50 (0.63 to 6.36)	3.44 (1.00 to 5.89)	0.05 (-3.82 to 3.71)	0.978
**Fracture rotation, degrees (95% CI)**				
X-axis (-anterior/+ posterior)	1.18 (-1.50 to 3.86)	1.60 (-0.67 to 3.87)	0.42 (-3.09 to 3.93)	0.814
Y-axis (-ante-/+ retroversion)	1.20 (-1.97 to 4.37)	2.75 (0.05 to 5.44)	1,55 (-2.61 to 5.71)	0.466
Z-axis (-varus/+ valgus)	0.16 (-1.34 to 1.02)	0.26 (-0.76 to 1.27)	0.41 (-1.14 to 1.97)†	0.602
**Post-weightbearing at 1 year**	**SHS (n = 8**)	**SHS with TSP (n = 12**)	**Mean difference**	**p-value**
Fracture translation mm (95% CI)				
X-axis (+ medial/- lateral)	2.65 (-4.46 to -0.84)	2.31 (-3.81 to -0.81)	0.34 (-2.01 to 2.68)	0.777
Y-axis (+ proximal/- distal)	6.45 (-8.88 to -4.01)	5.73 (-7.75 to -3.72)	0.71 (-2.45 to 3.87)	0.659
Z-axis (-anterior/+ posterior)	1.20 (-2.35 to -0.05)	1.46 (-2.42 to -0.51)	0.26 (-1.76 to 1.23)	0.727
Total translation (t3D)	7.81 (4.89 to 10.73)	6.87 (4.45 to 9.29)	0.94 (-4.73 to 2.85)	0.626
**Fracture rotation, degrees (95% CI)**				
X-axis (-anterior/+ posterior)	3.24 (0.52 to 5.96)	1.21 (-1.04 to 3.46)	2.03 (-5.56 to 1.50)	0.259
Y-axis (-ante-/+ retroversion)	4.01 (0.78 to 7.24)	2.34 (-0.34 to 5.01)	1.67 (-5.86 to 2.52)	0.434
Z-axis (-varus/+ valgus)	0.81 (-2.01 to 0.40)	0.82 (-1.82 to 0.18)	0.02 (-1.59 to 1.55)	0.981

* Linear mixed model.

† Mean z-rotation in opposite directions.

SHS, sliding hip screw; TSP, trochanteric stabilizing plate.

**Fig. 3:**
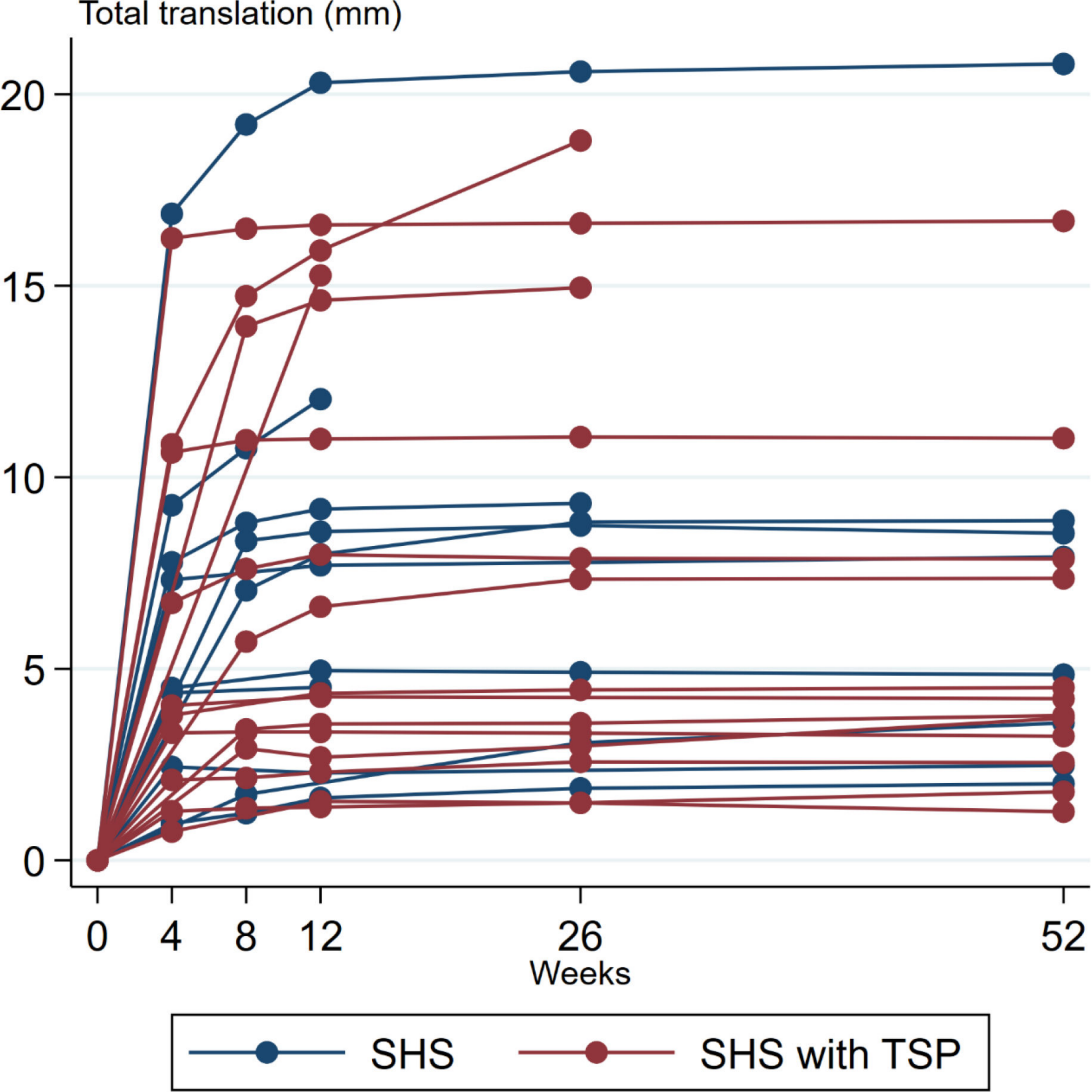
Graph showing total translation, in mm, for each patient (n = 29) included in the migration analysis. The total translation equals the sum of translation along the X-, Y-, and Z-axis (absolute values).

**Fig. 4:**
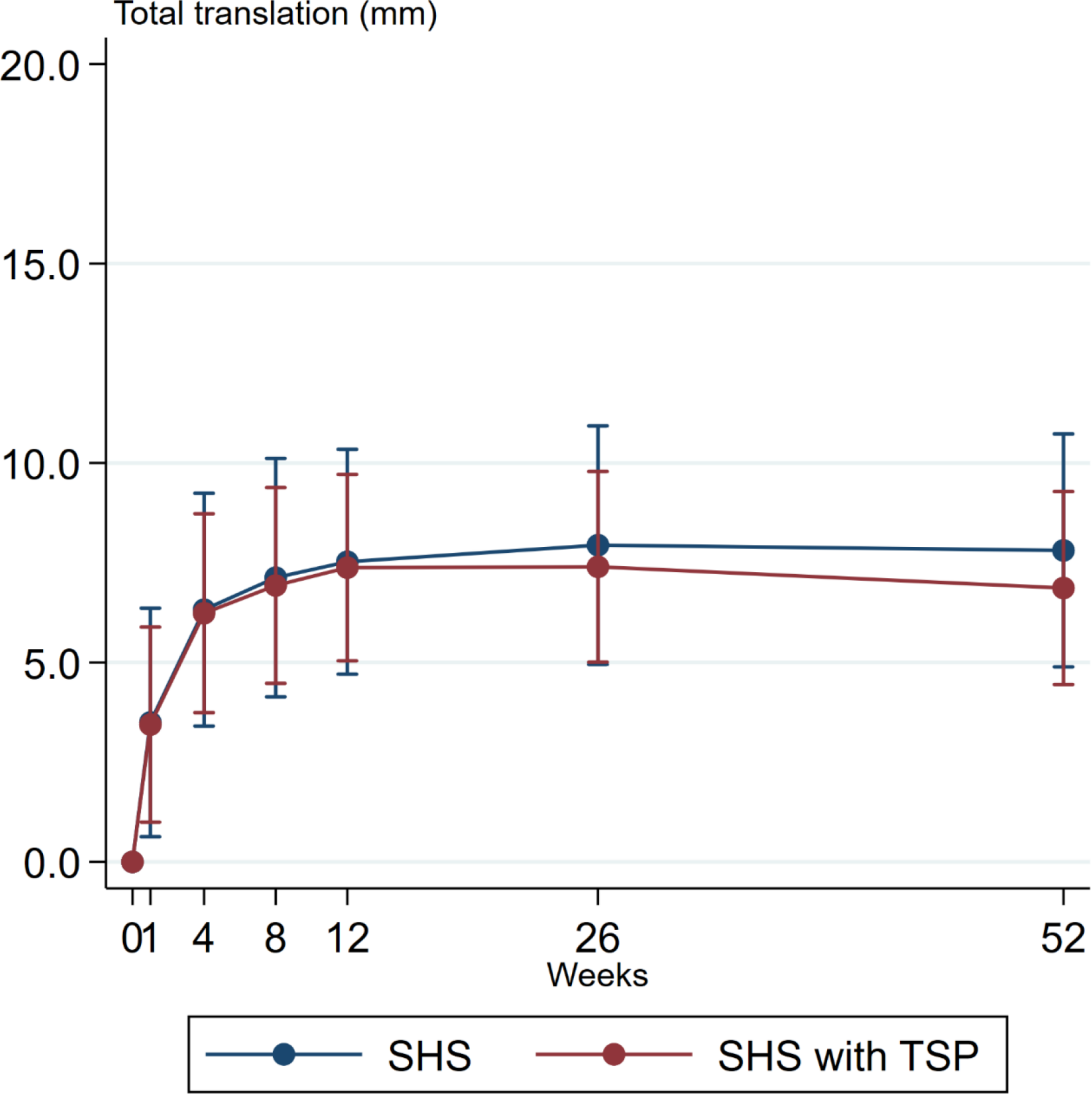
Graph showing mean total translation in mm, with 95% confidence interval, for both treatment groups. The total translation equals the sum of translation along the X-, Y-, and Z-axis (absolute values). No statistically significant differences were found at any time point.

**Fig. 5:**
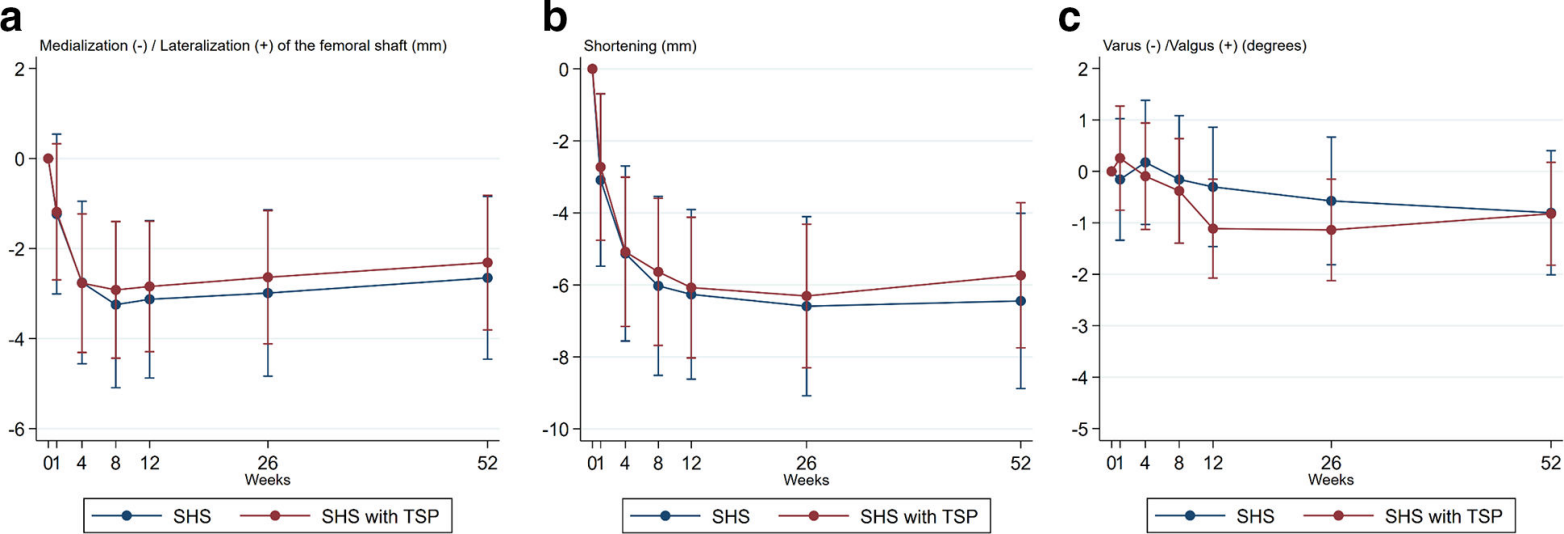
a) Graph showing mean translation (along the X-axis) in mm, with 95% confidence interval (CI), for both treatment groups. Negative values signify medialization of the femoral shaft relative to the head/neck fragment, while positive values mean lateralization of the shaft. b) Graph showing mean shortening (translation along the Y-axis) in mm, with 95% CI for both treatment groups. c) Graph showing mean rotation (around the Z-axis) in degrees, with 95% CI. Negative values signify fracture displacement towards varus, while positive values correspond to displacement towards valgus.

At one year, the total translation was 7.8 mm (95% CI 4.89 to 10.73) and 6.9 mm (95% CI 4.45 to 9.29) (Table III). No statistically significant differences were found between the 2 treatment groups at any time point in neither translation nor rotation (Figures 3 to 5).

Mean shortening at one year (Y-axis) was 6.5 mm (95% CI 4.01 to 8.88) in the SHS group and 5.7 mm (95% CI 3.72 to 7.75) in the TSP group. Mean lateralization (X-axis) of the proximal fragment relative to the shaft was 2.7 mm (95% CI 0.84 to 4.46) and 2.3 mm (95% CI 0.8 to 3.8). We found a mean rotation into varus of 0.8° (95% CI 2.01° varus to 0.40° valgus) in the SHS group with a corresponding mean rotation into varus of 0.8° (95% CI 1.82° varus to 0.18° valgus) in the TSP group (Table III).

There was one fixation failure after 26 weeks in the SHS group and two failures after three and 40 weeks, respectively, in the SHS with TSP group. All three patients were revised to total hip arthroplasty. There was no morbidity associated with implantation of the tantalum markers, and no need for additional surgery due to the markers.

Only minor differences in the clinical outcome measures were found at one year (Table IV), with comparable results at earlier time points (Supplementary Table ii).

**Table IV.** Clinical outcomes one year post-surgery.

Mean score, 95% CI	SHS	SHS with TSP	Difference	p-value*
Harris Hip Score	76 (67 to 86)	81 (74 to 88)	5 (-5 to 15)	0.325
EQ-5D VAS	67 (57 to 77)	76 (67 to 86)	9 (-4 to 23)	0.184
EQ-5D Index	0.78 (0.68 to 0.88)	0.75 (0.66 to 0.84)	0.03 (-0.17 to 0.11)	0.686
VAS pain	0.9 (0 to1.9)	1.1 (0.2 to 2.0)	0.2 (-1.2 to 1.5)	0.827
VAS satisfaction	8.1 (7.0 to 9.2)	8.8 (7.8 to 9.9)	0.7 (-0.8 to 2.3)	0.370
TUG test, sec (sec)	15.0 (11.4to 18.6)	14.3 (11.0 to 17.6)	0.7 (-5.6 to 4.2)	0.771

* Linear mixed model.

CI, confidence interval; EQ-5D, EuroQol five-dimension; SHS, sliding hip screw; TSP, trochanteric stabilizing plate; TUG, Timed Up and Go; VAS, visual analogue scale.

## Discussion

We found no clinically relevant or statistically significant differences between patients treated with an SHS with and without TSP for AO/OTA 31-A2 trochanteric fractures. The 95% CI of the mean difference of total translation (t3D) was less than±4 mm, which is presumably below a clinically relevant difference.^6^

The high measurement precision associated with RSA, enables analysis of fracture movement, and comparisons of different implants, with few patients, compared to studies using conventional radiographs.^8^ RSA is previously used in stable trochanteric fracture patterns investigating bone-implant movement,^17^ to compare fracture migration with IMN to SHS,^16^ and to evaluate the effect of cement augmentation in fractures instrumented with an SHS.^15^ In all of the above, RSA was proven a precise and viable method. To our knowledge, this is the first randomized RSA-study comparing postoperative fracture motion in unstable trochanteric fractures.

Haddon et al^8^ measured lag screw sliding on conventional radiographs in the only RCT published comparing SHS with and without TSP. The authors found a non-statistically significant mean difference in subsidence of 1 mm between the two groups for all fractures (Evans-Jensen types 3 to 5). No difference in fixation failure was shown. Comparable with Haddon et al,^8^ we found a statistically non-significant mean difference in total translation of 1 mm (95% CI -4.73 to 2.85) and a mean difference in shortening (translation along the Y-axis) of 0.71 mm in favour of the TSP group.

In a retrospective study, including both subtrochanteric fractures and trochanteric fractures with a broken lateral wall (Evans Jensen types 3 and 5), Madsen et al^28^ compared patients operated with SHS with a TSP to patients operated with either a SHS alone or an IMN. Less lag screw sliding was found in the TSP group compared to patients operated with an SHS alone. Unlike Madsen et al,^28^ we included only intermediate unstable fractures with an intact lateral column(AO/OTA type 31-A2 fractures). This could, at least partly, explain the different findings in our study. Since postoperative fracture movement in previous clinical papers is reported by lag screw sliding, a direct comparison to our results is not possible. However, finding only negligible movement along the Z-axis (anterior or posterior translation), the total translation in our data corresponds well with lag screw sliding.

Our study was not powered to detect statistically significant functional differences, but these were included to support the findings in the migration analysis. Akin to the migration analyses, only minor differences in clinical outcome were found between the treatment groups at all follow-ups.

A similar pattern of migration was found for both groups in all directions (Figure 5a to c), hence the TSP did not seem to influence the course of healing. As in Mattsson et al,^15^ the most pronounced movement in translation was found along the X- and Y-axis, as medialization of the femoral shaft and subsidence of the head/neck fragment. In rotation the movement was most prominent around the Y-axis towards retroversion and around the X-axis in flexion. This differs from the findings in the Mattsson et al^15^ study, where the movement was most apparent around the Z-axis towards varus. A possible explanation for our results is that the posteromedial comminution combined with the femoral neck anteversion might cause these fractures to move more into retroversion and flexion during the course of healing than previously appreciated. In addition, bone mineral density or the quality of reduction may influence the migration profiles. In consequence, possible differences between our study population and Mattsson et al^15^ might explain the discrepancies in rotation profiles.

In all cases, except one with later mechanical failure, the movement in both translation and rotation declined below the precision level at 12 weeks as an expression of fracture healing. No difference between the two treatment groups were found. These results are in line with both Bojan et al^17^ and Van Embden et al,^16^ who reported diminished fracture movement in the majority of cases after three months.

The main limitation of this study was missing data. Most of the fracture motion occurs within the first eight to 12 weeks postoperatively. Hence, it is a necessity to include early follow-up with stereographs when studying migration patterns, even though early follow-up of these elderly and fragile patients is challenging and some absence is ineluctable.

In addition, some RSA images had to be discarded from the analyses because they did not reach our predefined quality standards (CN, ME). The reasons for this included unstable tantalum markers in osteoporotic bone and the SHS plate interfering with the markers in the distal fragment or the cage. The standards for condition number and mean error of rigid body fitting are mainly defined by joint implant studies. In order to include more RSA examinations in the analysis it could have been worthwhile to increase CN and ME though at the cost of more uncertainty associated with our results. Hence, we chose to comply with the quality standards to ensure consistency in our migration data and to compare our result with previous RSA-publications. Due to limited availability of RSA technicians outside normal working hours and the need for early mobilization of the patients, we were left with 20 patients available for the analysis pre-/post-weightbearing (zero to one-week time point). As the missing data mainly were due to technical and logistical issues, we believe that data were missing at random, not producing selection bias.

Our precision was high, and comparable with Bojan et al.^17^ We wanted to be able to assess a 2 mm difference in total translation and calculated a need for eight patients in each treatment group. Despite patients lost to follow-up and missing RSA examinations we had more than eight cases available for analysis in each group both for the zero to one-week migration analysis and for the main outcome measure after one year (Figure 2). A sound correspondence between the observed data scatter and the statistical model was observed, further ensuring our data and associated results.

We did not address the emerging discussion of lateral wall thickness as its matter was not fully comprehended when planning this study. Data published mainly after we finished our protocol suggest that the TSP may be beneficial in in AO/OTA 31-A2 fractures with a thin lateral wall.^29,30^ If a potential benefit of the TSP was to be further explored, we would recommend adding lateral wall thickness in the inclusion criteria to ensure a sufficient number of fractures with critically thin lateral walls in both study groups or including only fractures with a compromised lateral wall (AO/OTA 31 A3) either in a large, RCT with clinical outcome measures or with a smaller study sample using CT-based migration analysis.

The TSP remains an implant without sufficient scientific backing. Apart from the discussion on lateral wall thickness cited above, there is to our knowledge no clinical data showing a benefit of the TSP in AO/OTA 31-A2 trochanteric fractures, either when comparing to SHS alone or with an intramedullary nail.^7^

The choice of implant for trochanteric fractures – or any treatment strategy – should not be based solely on a small RSA study, but rather on an array of functional outcome measures as well as potential surgical and medical complications. This study was underpowered for any other outcome than migration measured with RSA. However, we believe it contributes with valuable information for clinicians and researchers to better understand how and when (if at all) to use the TSP in clinical practice, and to plan for further studies with sufficient power to detect differences in clinical outcomes. In addition, we have produced new knowledge about how A2 fractures heal, and how the TSP may influence the course of healing.

In conclusion, finding only minor, non-significant differences in postoperative fracture motion between the two treatment groups, our data does not support routine use of the trochanteric stabilizing plate in AO/OTA 31-A2 trochanteric fractures.

**Take home message**

- Only minor, non significant, differences in postoperative fracture motion where found when comparing sliding hip screw with and without an additional trochanteric stabilizing plate (TSP) in AO/OTA 31-A2 fractures.

- Our results does not support routine use of the TSP in this fracture type.

## Data Availability

The datasets generated and analyzed in the current study are not publicly available due to data protection regulations. Access to data is limited to the researchers who have obtained permission for data processing. Further inquiries can be made to the corresponding author.
